# A Natural Plasmid Uniquely Encodes Two Biosynthetic Pathways Creating a Potent Anti-MRSA Antibiotic

**DOI:** 10.1371/journal.pone.0018031

**Published:** 2011-03-31

**Authors:** Daisuke Fukuda, Anthony S. Haines, Zhongshu Song, Annabel C. Murphy, Joanne Hothersall, Elton R. Stephens, Rachel Gurney, Russell J. Cox, John Crosby, Christine L. Willis, Thomas J. Simpson, Christopher M. Thomas

**Affiliations:** 1 School of Biosciences, University of Birmingham, Edgbaston, Birmingham, United Kingdom; 2 School of Chemistry, University of Bristol, Cantock's Close, Bristol, United Kingdom; University of Florida, United States of America

## Abstract

**Background:**

Understanding how complex antibiotics are synthesised by their producer bacteria is essential for creation of new families of bioactive compounds. Thiomarinols, produced by marine bacteria belonging to the genus Pseudoalteromonas, are hybrids of two independently active species: the pseudomonic acid mixture, mupirocin, which is used clinically against MRSA, and the pyrrothine core of holomycin.

**Methodology/Principal Findings:**

High throughput DNA sequencing of the complete genome of the producer bacterium revealed a novel 97 kb plasmid, pTML1, consisting almost entirely of two distinct gene clusters. Targeted gene knockouts confirmed the role of these clusters in biosynthesis of the two separate components, pseudomonic acid and the pyrrothine, and identified a putative amide synthetase that joins them together. Feeding mupirocin to a mutant unable to make the endogenous pseudomonic acid created a novel hybrid with the pyrrothine via “mutasynthesis” that allows inhibition of mupirocin-resistant isoleucyl-tRNA synthetase, the mupirocin target. A mutant defective in pyrrothine biosynthesis was also able to incorporate alternative amine substrates.

**Conclusions/Significance:**

Plasmid pTML1 provides a paradigm for combining independent antibiotic biosynthetic pathways or using mutasynthesis to develop a new family of hybrid derivatives that may extend the effective use of mupirocin against MRSA.

## Introduction

**Figure 1:**
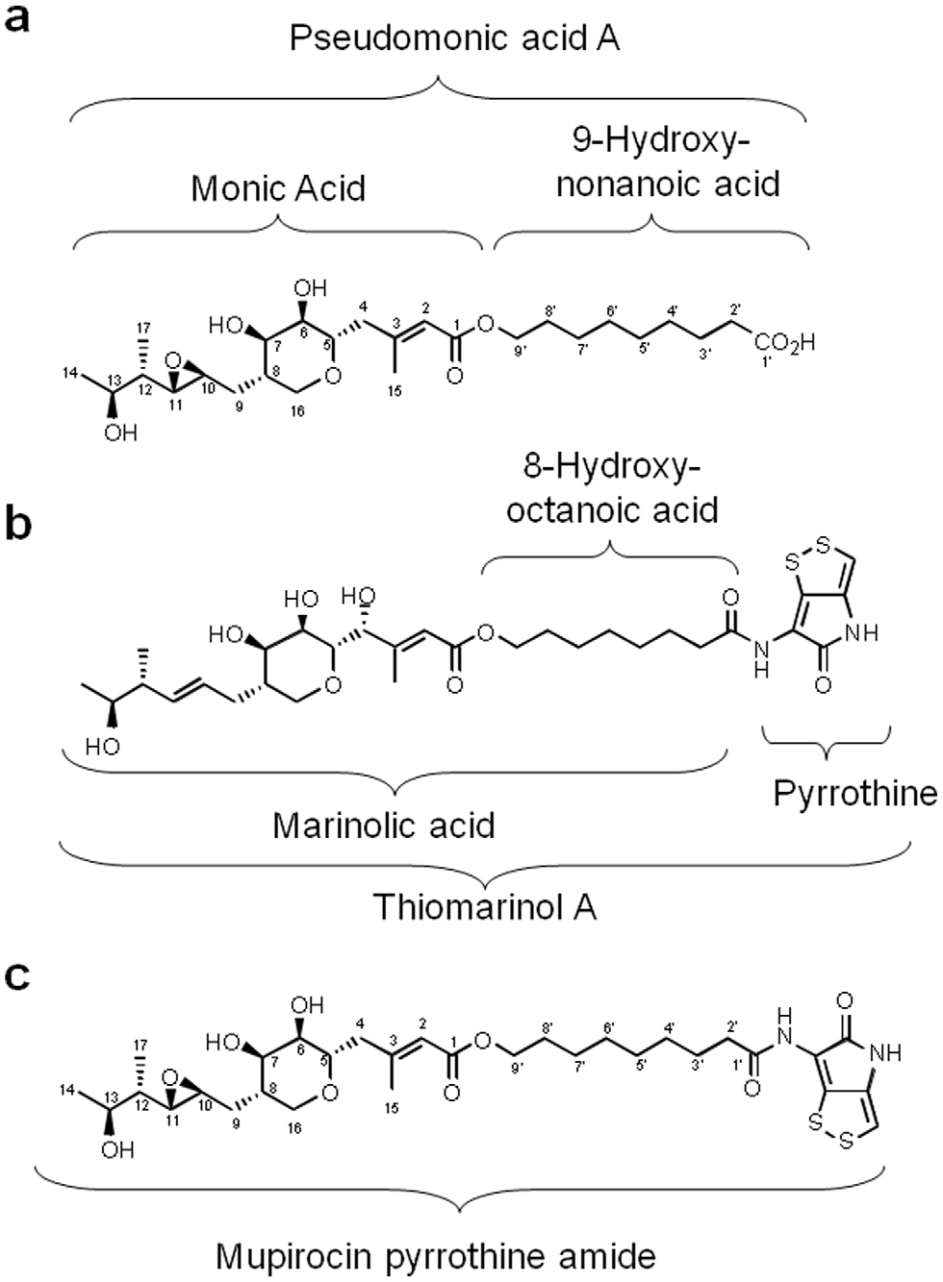
Chemical structures of A pseudomonic acid A (mupirocin) and B thiomarinol A. Holomycin is the amide *N*-acetylpyrrothine. In addition to thiomarinol, producer-bacteria generate a variety of fatty acyl pyrrothine analogues. The new hybrids created consist of pseudomonic acid A **A** with pyrrothine joined via an amide bond as in **B** to create the mupirocin pyrrothine amides **C**.

The inexorable rise of antibiotic resistance in clinically important bacteria makes it vital not only to identify new classes of antimicrobial compounds but also to develop new derivatives of existing compounds that can extend their effective use. Thiomarinols [1]–[4], produced by marine bacteria, form a novel family of natural compounds with potent antimicrobial activity. They consist of two components each related to independent antibiotics: the clinically important anti-methicillin resistant *Staphylococcus aureus* (MRSA) antibiotic mupirocin in which a polyketide moiety, monic acid, is esterified by an unusual fatty acid component, 9-hydroxynonanoic acid [5], [6]; and a pyrrothine moiety, previously found in the antibiotic holomycin [7] and related compounds. In thiomarinol the pyrrothine is attached via an amide linkage to an 8-hydroxyoctanoic acid side-chain in the mupirocin-like component, called marinolic acid by analogy with mupirocin's more generic name, pseudomonic acid (Figure 1).

The mupirocin biosynthetic (*mup*) gene cluster from the soil and plant root-associated bacterium *Pseudomonas fluorescens* was sequenced some time ago [5] and many of the key steps of the biosynthetic pathways have been worked out over the last 10 years [6]. When this project started the genes responsible for holomycin production by the producer organism *Streptomyces clavuligerus* [8] were not known but very recently the identification of the gene cluster and the analysis of key steps in the pathway have been described [9]. Mupirocin is particularly effective against Gram-positive bacteria [6] whereas thiomarinols are also effective against many Gram-negative species [1]. This increased potency may be due to: increased uptake, since the outer membrane is thought to be a barrier that protects Gram-negative bacteria; the targeting of more than one cellular function, since the pyrrothine moiety in the form of holomycin and thiolutein inhibits RNA synthesis [7] rather than protein synthesis which is the ultimate target of mupirocin; or increased activity against the normal target for mupirocin, isoleucyl-tRNA synthetase (IleS) [10].

The purpose of this work was to determine what mechanism brings together the products of these two pathways and establish whether this reveals a strategy for creating new hybrid molecules that may have useful biological activities. The results reveal a novel plasmid that is devoted almost entirely to carriage of these two biosynthetic pathways and indicate not only new mutasynthesis pathways but a paradigm for generating new strains that combines the products of different pathways and creates more potent bioactive molecules.

## Results

### The thiomarinol biosynthetic genes are encoded on a plasmid

**Figure 2:**
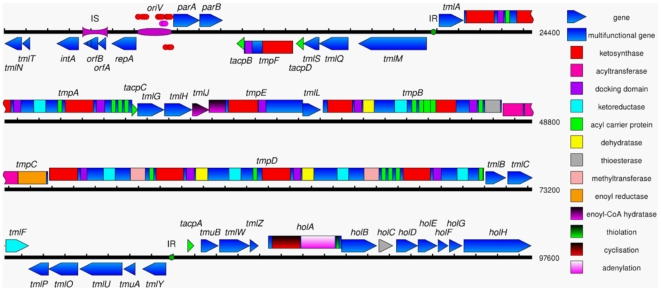
Map of pTML1 showing predicted protein-coding sequences drawn above or below the DNA line to indicate direction, with predicted biosynthesis domains colour-coded as listed in the key. Modules can be identified by segments of megaproteins running from a KS (red) to an ACP (green). Putative protein binding sites are shown as red and purple discs (in the replication origin, *oriV*) and green discs (‘IR’ for inverted repeat, associated with the biosynthetic cluster promoter regions and likely to be transcriptional regulator binding sites). Like the mupirocin biosynthetic genes the thiomarinol synthases belong to the trans-AT synthases that encode a separate Acyl Transferase while linked to each KS domain is an adjacent “docking domain” consisting of incomplete motifs from Acyl Transferases [11] that may facilitate or regulate acyl transfer [12].

**Figure 3:**
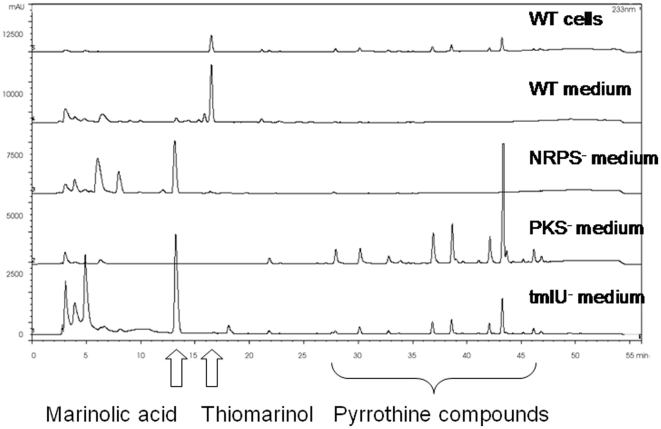
HPLC analysis of products from wild type *Pseudoalteromonas rava* SANK 73390 and mutant derivatives. In the wild type the majority of thiomarinol is present in the medium while significant levels of aliphatic pyrrothines are associated with the cells. The results show that: in the KS^−^ mutant, only the acyl pyrrothines are produced; in the NRPS^−^ mutant only marinolic acid as produced; and that in the *tmlU* mutant, both marinolic acid and acyl pyrrothines are produced by thiomarinol itself is not produced.

To identify the genes for thiomarinol biosynthesis we sequenced total DNA from the producer organism, *Pseudoalteromonas sp* SANK 73390. One of the 273 contigs obtained (approximately 97 kb) encoded many genes with similarity to those of the *mup* cluster (Figure 2). As part of preliminary work we had used degenerate primers, designed on the basis of conserved segments of the mupirocin ketosynthase (KS) coding regions, to amplify putative thiomarinol KS sequences as described in Materials and Methods. This had yielded a number of products which were sequenced and one of these which matched part of the largest polyketide (PKS) gene (*tmpD*) was used for suicide mutagenesis using vector pAKE604 [13]. Mutants showed reduced antibacterial activity and LCMS analysis confirmed the loss of thiomarinol production, but continued production of pyrrothine-containing compounds (Figure 3).

Further analysis of this contig showed that it constitutes a circular plasmid which we designate pTML1 (Figure 2). The evidence for it being a circular plasmid is as follows. First, the initial annotation identified the 5′ and 3′ ends of the same gene at opposite ends of the 97 kb contig and further analysis identified reads across the apparent gap which is artificial, created by the fact that the standard assembly software does not seek to create circles. Furthermore, we also considered the remote possibility that the single copy of the putative Insertion Sequence (IS) might actually represent the insertion site of a larger segment (or segments of a linear plasmid) flanked by identical copies of the same element. To test this possibility we performed PCR with primers described in Materials and Methods that hybridised to the unique sequences on either side of the putative IS. A clean product of the expected size (3822 bp) was observed, confirming the absence of an additional segment. Second, one orf in this contig encodes a putative DNA replication initiator (Rep) protein similar to that of the megaplasmid-like Chromosome II of *Pseudoalteromonas haloplanktis* TAC125 [14] (CR945247: 635,328 bp) and two adjacent orfs encode proteins similar to plasmid partitioning proteins from the same source. Third, using sucrose selection against the pAKE604:*tmpD* suicide mutant (that is, sucrose sensitivity due to levansucrase encoded by pAKE604) we could isolate white colonies (indicating a loss of production of the yellow pigmented pyrrothine moiety of thiomarinol) where loss of the whole element was confirmed by multiple PCR reactions spread across the plasmid including the *rep* gene. Thus pTML1 is not essential for SANK 73390 to survive.

### Thiomarinol biosynthesis depends on two independent pathways

**Figure 4:**
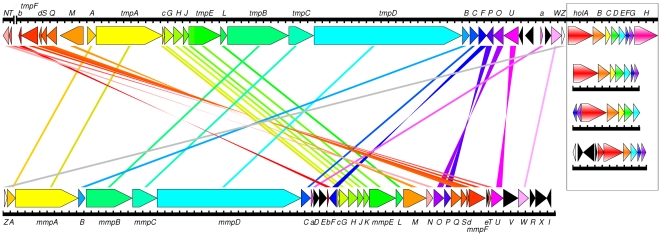
Comparison of the organisation of the thiomarinol gene cluster (upper line) with the mupirocin biosynthesis gene cluster from *Pseudomonas fluorescens* (lower line) and (boxed, on right) with related NRPS clusters from (top to bottom) *Yersinia ruckeri* ATCC29473, *Streptomyces clavuligerus* ATCC27064 and *Photorhabdus asymbiotica* ATCC43949. Lines connecting orfs are simply to help identify equivalent genes and do not indicate the degree of relatedness. A full map of pTML1 is shown in Figure 2. *macpE* (labelled “e”), which is critically missing from the thiomarinol cluster, lies between *mmpF* and *mupT*.

After finishing, the sequence of pTML1 is 97,600 bp with a G+C content (43.2%), slightly higher than the average for all the genomic DNA (40.6%) but contrasting with the *P. fluorescens* mupirocin biosynthetic cluster (G+C = 56%) [5]. Annotation showed forty five orfs (Figure 2 and Table S1; Accession number FN689524). Five orfs, occupying only 7.6 kb, were typical of mobile DNA (replication, partitioning, transposition and integration) but showed no evidence of conjugative transfer functions. Twenty seven orfs encode products expected for an essentially complete mupirocin biosynthetic cluster producing marinolic acid (Figure 1). The gene order for the type I polyketide synthase (PKS) genes (*tmpA* to *tmpD*) is similar to the *mup* cluster [5] (Figure 4). Minor differences in the functions present compared to the *mup* cluster are an extra ACP in the third module of TmpD, an extra ACP in the last module of TmpA and two extra ACPs in TmpB, one in a group of four and one following an extra KS to create a second module in this protein (Figures 2 and 4). Multiple ACPs in the mupirocin cluster have been found to generally increase pathway throughput rather than being essential [15] By contrast, the tailoring genes are significantly rearranged (Figure 4) the whole gene set being split into at least five transcriptional units distinguished by direction of transcription and interruption by the backbone functions of *rep*/*par* and transposition functions (*tmlT* to *tmlN*, *tmlM* to *tacpB*, *tmlA* to *tmlF*, *tmlY* to *tmlP* and *tacpA* to *holH*; Figure 2). Although the low G+C content of the DNA makes it quite difficult to predict promoter sequences, closely related inverted repeats are found in the spaces between the divergent *tmlM* to *tacpB*/*tmlA* to *tmlF* and *tmlY* to *tmlP* and *tacpA* to *holH* units and are likely candidates for operator sequences through which the genes are regulated, although no obvious regulatory protein was found in the plasmid.

**Figure 5:**
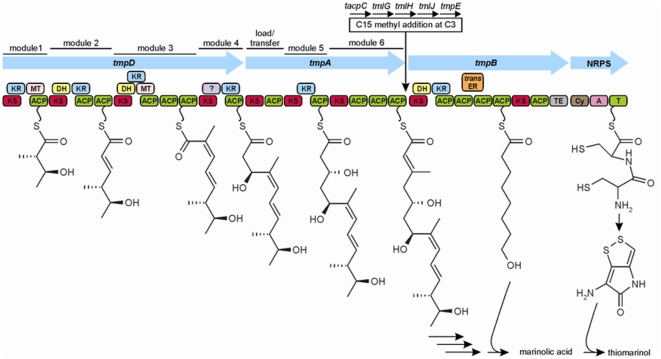
Predicted biosynthetic scheme for thiomarinol biosynthesis showing the roles of TmpD (modules 1 to 4)/TmpA (modules 5 and 6) for monic acid, TmpB for 8-hydroxyoctanoic acid and HolA/NRPS for pyrrothine. A detailed scheme for biosynthesis of the pyrrothine can be found in ref [9]. Individual functions in multifunctional proteins are shown by the colour-coded blocks: KS, Ketosynthase; ACP, Acyl Carrier Protein; KR, Ketoreductase; DH, Dehydratase; ER, Enoyl Reductase; MT, Methyl Transferase; TE, Thioesterase; C, Condensation; A, Aminoacyl Adenylation Domain; T, Thiolation Domain or Peptidyl Carrier Protein.

The absence of mAcpE, implicated in the last steps of pseudomonic acid production [16], but an extra module (KS and ACP) in TmpB, suggests that all late tailoring steps may happen on TmpB (Figure 5). There is a clear conservation of the PKS and associated parts of the biosynthesis clusters for mupirocin and thiomarinol (Figure 4). However, a high degree of sequence divergence between the predicted products of each ORF (the level of sequence identity varies from approximately 40% to 60% identity in amino acid sequence alignments, although the identity is locally higher in functionally constrained regions, Table S1) is observed. This raises intriguing questions about whether there will be functional cross-complementation between the pathways, an issue that will be investigated in a future publication.

A particularly exciting feature of the gene cluster was a group of seven orfs with similarities to several putative non-ribosomal peptide synthetase (NRPS) gene clusters which until very recently had no assigned product(s). These include clusters from *Streptomyces clavuligerus* (genome sequence of strain ATCC 27064, accession numbers EDY50341 and ADWJ01000000 [17]) that makes holomycin [7]–[9] and *Photorabdus assymbiotica* [18] and *Yersinia ruckeri* ATTC43949 [19] that synthesize related compounds [20] (Figure 4). We therefore designated these genes *holA* (NRPS), *holB* (oxidoreductase), *holC* [thiolesterase), *holD* (dehydrogenase), *holE* (acyltransferase), *holF* (oxygenase), and *holG* (decarboxylase). Functional analysis of the homologous genes from *S. clavuligerus* has recently been reported, confirming their roles but leaving significant questions [9]. For example, the encoded NRPS (HolA), which possesses active site amino acids consistent with selectivity for cysteine [21], encodes only single adenylation, thiolation (peptidyl carrier protein, PCP), and condensation domains while a dipeptide would formally require two adenylation and two PCP domains. Since the pyrrothine should start with formation of a cysteinyl-cysteine dipeptide, HolA may be an iterative NRPS as in siderophore biosynthesis [22] and could possibly work as a dimer. The function of HolH, which does not have a counterpart in the related clusters, is not known. An overview of the predicted pTML1 thiomarinol biosynthetic pathway is shown in Figure 5.

To confirm the identity of the genes encoding pyrrothine production an internal segment of *holA* was used for suicide mutagenesis, producing mutants lacking yellow pigment. LCMS of WT bacteria showed thiomarinol A (*m/z* 640 Da) in both whole cells and supernatant whereas the *holA* (NRPS) mutant produced only marinolic acid (*m/z* 486 Da). Similarly the *tmpD* (PKS) mutant produced only yellow pigmented pyrrothine material (Figure 3). This confirms that marinolic acid and the pyrrothine can be made separately. TmlU shows similarity to SimL (Figure S1) that creates an amide linkage during biosynthesis of the antibiotic simocylinone [23], [24] as well as NovL which plays a similar role in novobiocin biosynthesis [25]. A mutant with an in-frame deletion in *tmlU* made both marinolic acid and pyrrothines but no thiomarinol (Figure 3) strongly suggesting a role for TmlU in joining them together.

### Mutants defective in one or other pathway can use exogenous substrates

To test for cosynthesis we co-fermented the Δ*tmpD* and Δ*holA* mutants. LCMS analysis confirmed that production of thiomarinol was restored. Addition of marinolic acid to the Δ*tmpD* mutant also restored thiomarinol production. Furthermore, addition of pseudomonic acid A to the Δ*tmpD* mutant gave rise to two novel pyrrothine amides (Figure 1c) indicating that mutasynthesis is possible. Feeding with a range of alternative substrates reported fully elsewhere [26] showed that alternative pseudomonic acid metabolites produced by *P. fluorescens* or amines (for example anhydroornithine) could be incorporated by Δ*tmpD* and Δ*holA* mutants respectively confirming the potential to create new families of hybrids.

### Thiomarinol can overcome mupirocin resistance

**Figure 6:**
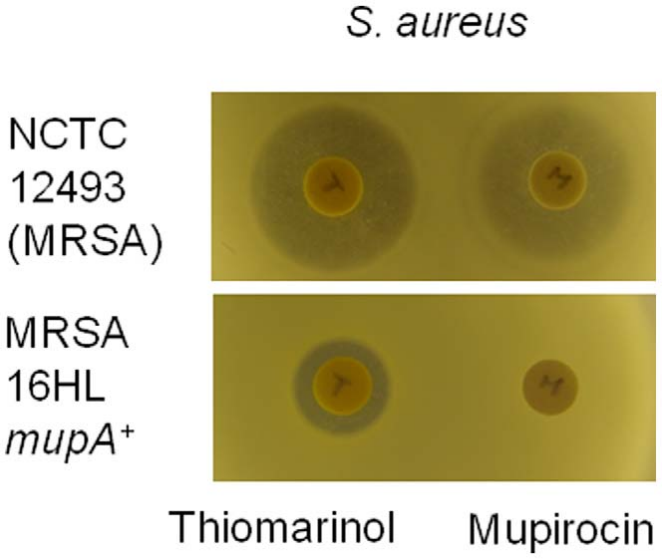
Bioassay comparing the effects of thiomarinol and mupirocin against Methicillin Resistant *Staphylococcus aureus* (MRSA) and High level Mupirocin Resistant MRSA. The loaded amount of thiomarinol was calculated as 25 µg, and same amount of PA-A was spotted onto the paper disc “M”.

Plasmid-free segregants of SANK 73390 were sensitive to thiomarinol confirming that resistance requires pTML1. The *tmlM* gene encodes a putative isoleucyl tRNA synthetase related to MupM that prevents suicide in the mupirocin producer [5], [27] but no additional gene(s) conferring resistance to the pyrrothine element was identified. Expression of *mupM* and *tmlM* in *E. coli* under the control of the *tac* promoter and determination of thiomarinol resistance (by both plate bioassay and minimal inhibitory concentration, MIC, determination) with appropriate controls showed that *mupM* confers slight resistance to thiomarinol (MIC = 1 µg ml^−1^ compared to 0.5 µg ml^−1^ for the empty vector) while *tmlM* confers high level resistance (MIC≥16 µg ml^−1^), suggesting that thiomarinol targets only isoleucyl tRNA synthetase. Thiomarinol was therefore tested for its ability to inhibit growth of methicillin resistant *Staphylococcus aureus* (MRSA) that had acquired high level resistance to mupirocin. A plate bioassay showed that clearly thiomarinol could inhibit growth of this strain although the clearing zone was considerably smaller than with the mupirocin sensitive strain (Figure 6). We checked by PCR that this strain carries the *S.aureus mupA* gene, that encodes an isoleucyl tRNA synthetase conferring high level mupirocin resistance, and then cloned and expressed it in *E. coli* from a context identical to that for *mupM* and *tmlM*. It also conferred resistance to mupirocin in *E. coli* (MIC = 50 µg ml^−1^) and gave partial resistance to thiomarinol (MIC = 2 to 4 µg ml^−1^) slightly higher than that for the *mupM* gene but was clearly inhibited at concentrations that were not inhibitory for bacteria carrying *tmlM*. Thus essentially complete resistance to thiomarinol can be conferred by an altered *ileS* gene (*tmlM*) while the mupirocin resistant *ileS* genes (*mupM* and *mupA*) only confer partial resistance.

Biological activities of a selection of the new compounds identified in this work [26] were compared by plate bioassay and minimum inhibitory concentration in liquid culture with *E. coli* and *S. aureus* (MRSA) as indicator strains. Both thiomarinol and the novel mupirocin pyrrothine amides created by feeding mupirocin as the standard mixture of pseudomonic acids, inhibited growth of both species although *S. aureus* was more sensitive (MIC≤30 ng ml^−1^ compared to 0.5 µg ml^−1^). Marinolic acid inhibited *S. aureus* (MIC = 1 µg ml^−1^) but less well than mupirocin (MIC = 0.125 µg ml^−1^) confirming that the added pyrrothine is responsible for the increased potency of thiomarinol. Individual fatty acylpyrrothines isolated from *Pseudoalteromonas sp* SANK 73390 had no activity against *E. coli* and only weak activity against *S. aureus* [26]. Against MRSA carrying *mupA*, only the pyrrothine amides were active and thiomarinol (MIC = 8 µg ml^−1^) was slightly more active than the mupirocin pyrrothine amide (MIC = 16–32 µg ml^−1^). The partial resistance of mupirocin-resistant MRSA to thiomarinol is consistent with the partial resistance that *mupA* confers to thiomarinol in *E. coli*.

## Discussion

Although a variety of mechanisms allow plasmid-bearing bacteria to kill their neighbours [28] pTML1 uniquely dedicates essentially all of its large genome to making a single family of hybrid molecules which have potent antibiotic activity against both Gram-positive and Gram-negative bacteria. It may thus function as a way of providing an advantage for bacteria carrying the plasmid but the density of plasmid positive bacteria required to achieve this effect is not yet known. Antibiotic biosynthesis clusters on other plasmids are generally found as just one of a number of functions. The genes for methylenomycin occupy only about 20 kb on the 356 kb megaplasmid SCP1 in *Streptomyces coelicolor* A3 [29] while production of tropodithietic acid occupies about 10 kb on plasmids ranging from 60 to 130 kb in marine *Roseobacter* species [30]. The 211 kb plasmid pSLA2-L from *Streptomyces rochei* carries two antibiotic clusters, for lankamycin and lankacidin, among four secondary metabolite gene clusters which occupy 75% of the plasmid [31]. More recently a 1.8 Mb linear plasmid from *Streptomyces clavuligerus* was found to carry numerous secondary metabolite biosynthetic clusters [32]. A plasmid location might promote spread of these genes between hosts but surprisingly there is no evidence of conjugative transfer genes in pTML1. It is also surprising that, although there are putative operator sequences (inverted repeats shown in green, Figure 2) in the divergent promoter regions, there are no obvious candidates for regulatory proteins in the plasmid (Table S1) suggesting that regulation may be dependent on chromosomal genes and posing a potential problem for the plasmid if it were to transfer to a host that was not able to provide this function. This is doubly surprising since the mupirocin genes in *P. fluoresens* have their own quorum regulation cassette [13]. While these gene sets appear to have been acquired from different ancestors neither the average G+C content of the two gene sets nor the codon usage give any indication of this, suggesting that the genes have evolved together for some considerable time.

Our results confirm the hypothesis that both components of thiomarinol, the pseudomonic acids similar to mupirocin and the pyrrothine similar to holomycin, are synthesised by gene sets related to those that synthesise these molecules from organisms that make them independently [5], [9]. Detailed comparison of the pseudomonic acid and marinolic acid biosynthesis genes reveals a number of interesting features apart from the obvious rearrangement of gene order (Figure 2).

First, there is no evidence for regulatory genes as part of the thiomarinol gene cluster and the putative operators in likely promoter regions do not show obvious similarity to the Lux boxes found in the *mup* cluster [13], [33] so we can not make any predictions about mechanism, if any, by which expression is regulated.

Second, alignments of TmlA not only identify MupA but also OnnC and PedJ proteins from the onnamide and pederin pathways in which they are proposed to introduce hydroxyl groups at C7 [34], [35]. Since the location of *mupA*/*tmlA* immediately upstream of *mmpA*/*tmpA* is conserved in both clusters we hypothesise that it is responsible for the C6 hydroxylation of monic acid and that this occurs while it is attached to the non-elongating module at the start of MmpA/TmpA [5].

Third, the hydroxymethylglutarate CoA synthase (HCS) cassette which is responsible for insertion of the C15 methyl group [36] lacks an independent gene encoding an homologue of MupK, the second hydratase (responsible for decarboxylation, the last step in the pathway), although this function seems likely to be provided by TmpE whose N-terminus aligns with MupK. In the *mup* cluster *mupK* and *mmpE* lie one after the other so a simple gene fusion could be responsible for this arrangement. However, in the kalamanticin/batumin cluster the Type I PKS Bat3 has a similar conjunction of a hydratase followed by a KS at the junction of modules 9 and 10 as part of a much larger type I PKS protein [37]. Significantly, this is where the second of four methyl insertions by the HCS cassette occurs. However, since the sequence identities with MupK (60%) and BatE (57%), its equivalent in the kalamanticin/batumin cluster, are higher than with the hydratase segment in Bat3 it seems more likely to have arisen by the proposed fusion than direct descent as a fused protein from a common ancestor with Bat3. An additional inference from this fusion, based on the assumption that most parts of the biosynthetic factory are tethered in an orderly assembly, is that the action of the KS and any additional functions of TmpE may normally be performed at the same stage of biosynthesis that the HCS cassette works (i.e. at completion of the monic acid backbone).

Fourth, the only gene in the *mup* cluster that does not have an equivalent in the thiomarinol biosynthetic cluster is *macpE*, that is essential for pseudomonic acid A production but not pseudomonic acid B [16]. In the *mup* cluster we have proposed that MupU is responsible for transferring an intermediate to mAcpE where it is processed by MupO,V,C and F [33]. However, TmpB has an extra KS and ACP that MmpB does not have, while TmlU clearly has a role that is different from, or additional to, the role of MupU in transferring an intermediate to mAcpE suggesting the possibility that the final processing to produce thiomarinol happens on TmpB before release by the action of the TmpB TE. This could mean that there is less potential for leakiness (release before completion of all biosynthetic steps) at this stage of biosynthesis which might explain why thiomarinol G, the equivalent of pseudomonic acid B (hydroxylated at C9; Figure 1), is hardly detectable (AM, unpublished). Work to test this hypothesis is underway.

The identification of a single thiomarinol resistance gene, *tmlM*, encoded on pTML1 and the finding that this gene is sufficient to confer high level (essentially complete) thiomarinol resistance indicated that the enhanced activity of thiomarinol compared to mupirocin is most likely due to an increased ability to bind to and inhibit isoleucyl tRNA synthetase rather than activity against a second target. This also indicates that the ability of thiomarinol to inhibit the growth of bacteria carrying the mupirocin resistant isoleucyl tRNA synthetases encoded by either *mupM* from the *P. fluorescens* mupirocin-producer strain NCIMB10586 or *mupA* from *S. aureus* is likely to be due to this increased potency against these IleS enzymes rather than the inhibition of a second target. This is also consistent with the finding that the potency of thiomarinol is destroyed by hydrolysis of the amide bond which indicates that the molecule must remain intact to exert its effect. An alternative explanation, that the hybrid is hydrolysed to its component parts after entry and that these work separately, is not consistent with the data. The results therefore provide justification for exploration of alternative additions to the carboxylic acid end of pseudomonic acid in order to identify ways to overcome mupirocin resistant enzymes. Interestingly, MupM is not the closest relative of TmlM, there being many other homologues from other prokaryotes as well as from higher eukaryotes that are much closer in sequence. Whether this indicates selective pressure or horizontal gene transfer and recombination is not clear.

This is the first documented example of independent pathways to known antibiotics combining to create a hybrid, although antibiotics simocyclinone [38] and onnamide [35] are built from sub-pathways. Our data show that the added pyrrothine increases potency of marinolic/pseudomonic acid against isoleucyl tRNA synthetase, overcoming resistance to mupirocin in contrast to chemical modifications explored previously [39]. We identified a gene *tmlU* that is required for formation of the amide. The product of this gene belongs to a growing family of amide synthases [22]–[24] that provide important potential for creating diverse hybrid molecules [40]. It may allow second site interactions that strengthen binding as proposed for simocylinone with DNA gyrase[41]. In combination with the crystal structure of isoleucyl tRNA synthetase with mupirocin [42] our data provide the basis for generating new families of compounds that may be useful against MRSA.

## Materials and Methods

*Pseudoalteromonas* sp SANK 73390 and *E. coli* strains was grown on Marine broth/agar [1] at 23°C, *Escherichia coli* DH5α and S17-1 were grown on L Broth or L agar at 37°C [43] and *Staphylococcus aureus* NCTC 12493 and 16HLmupR1 were grown on Mueller Hinton II broth/agar at 37°C. Cloning vector pGEM-T (www.promega.com) and suicide plasmid pAKE604 [13] were described previously.

Genomic and plasmid DNA isolation, manipulation, transformation and analysis were carried out by standard procedures [43]. The genome sequencing was performed at the Liverpool Microarray Facility at University of Liverpool using a 454 Titanium FLX kit and hardware. Automated assembly into contigs was achieved using 454 Newbler assembler software and finishing was directed by Phred/Phrap/Consed software, PCR with primers from Sigma and sequencing in the University of Birmingham Genomics lab using an ABI 3700 capillary sequencer. Sequence analysis and annotation used Artemis/ACT software as well as http://www.nii.res.in/nrps-pks.html which are specific for PKS/Non Ribosomal Peptide Synthetase functions. To confirm that the putative IS element is a single copy and does not represent a segment flanked by two copies PCR was performed with primers that hybridise in the unique segments on either side (5′ –TCCCTTGTGCTAGTGTCATAG -3′ and 5′-AGGCTTGAGTCAGATTAAGTCT-3′).

Gene knockouts were performed as described previously using suicide plasmid pAKE604 [13] into which we cloned selected segments from the target genes, mobilising to SANK 73390 via *E. coli* strain S17-1. For the KS (*tmpD*) knockout a 1 kb segment corresponding to position 5182 to 6158 in *tmpD* (KS-D2) which had been amplified with degenerate primers (5′ - ATGGAYCCBCAGSARCGYYTGTT -3′ and 5′ - TTYGGBKYCGGCGGKRCSAAYGC -3′) was used. For the NRPS knockout a 480 bp segment corresponding to positions 1509 to 1990 using specific primers (5′- CCA**GAATTC**GATGCAAATGCTAGGCT -3′ **incorporating a EcoRI site and 5′- AGCTAAAGCTT**CTAGTTCTGCAACTC -3′**** incorporating a HindIII site) was used. For the *tmlU* knockout an in-frame deletion was created by using PCR to generate upstream (5′- TTTAG**TCTAGA**TAGGCGCAACCTT G -3′ **incorporating an XbaI site and 5′- CGGGAAAACG**CTGCAG**AAATGGATG -3′** incorporating a PstI site) and downstream (5′- CGTTTA**CTGCAG**TGCTCGCTGAGC -3′ **incorporating a PstI site and 5′- ATAGCCCT**GTCGAC**CGTACCCAA -3′** **incorporating a SalI site) arms that were joined via the common PstI site and cloned into pAKE604.**

Antibiotic plate bioassay was performed as described previously [5]. Expression constructs for *mupM*, *tmlM* and *mupA* (from *S. aureus*) were constructed based on *tac* promoter expression vector pJH10 [5]. In addition, thiomarinol was extracted from 2 day cultures of SANK 73390 and dried onto filter paper discs that were placed on bacterial lawns. HPLC and mass spectrometric analysis of bacterial products were performed as described elsewhere [44].

## Supporting Information

Figure S1 **Alignment of the amino acid sequences of TmlU, SimL (AAG34183) and NovL (AAF67505).**
(TIF)

Table S1 **Predicted gene products of pTML1.**
(DOC)
