# Correction: A Natural Plasmid Uniquely Encodes Two Biosynthetic Pathways Creating a Potent Anti-MRSA Antibiotic

**DOI:** 10.1371/journal.pone.0116036

**Published:** 2014-12-15

**Authors:** The PLOS ONE Staff

In Figure 2, the labels for the genes *tmlY* and *tmuA* are incorrectly switched. Please view the correct Figure 2 here.

**Figure 2:**
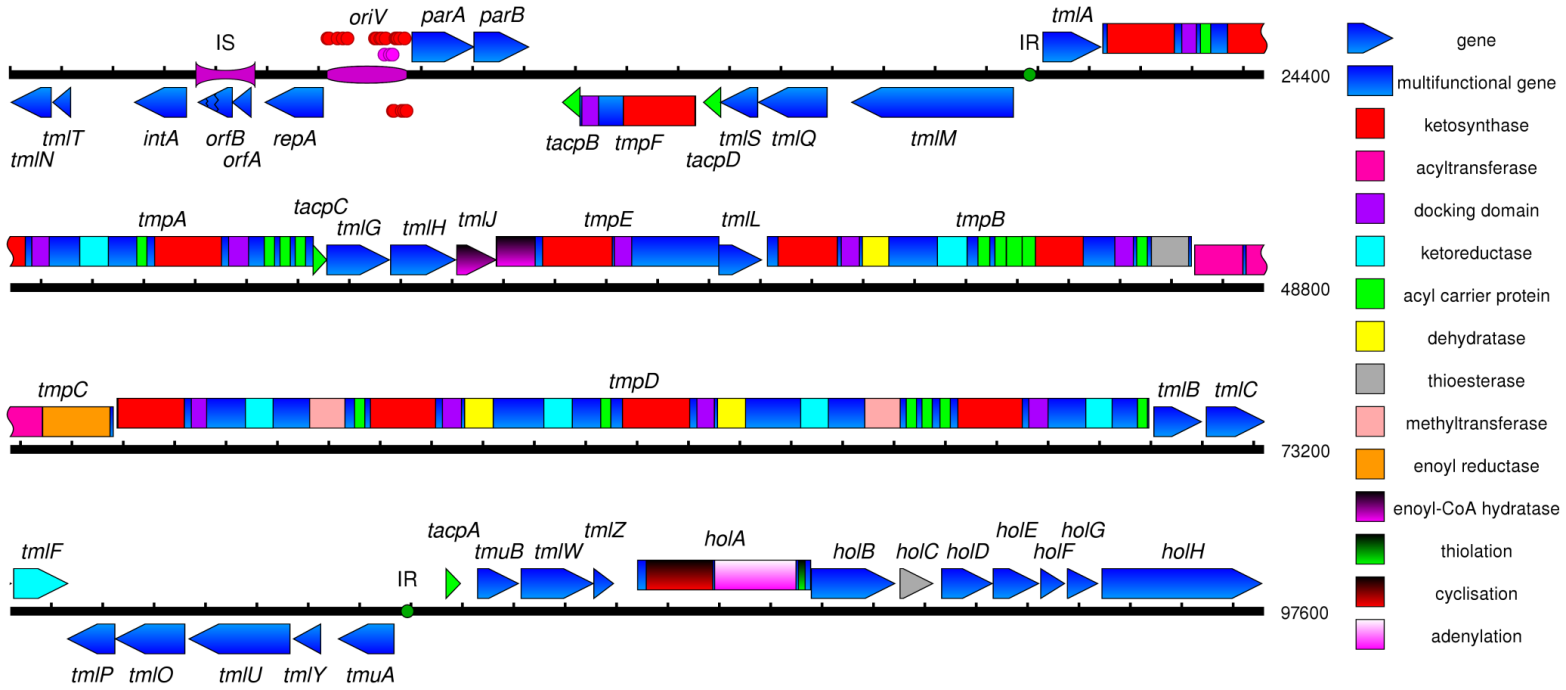
Map of pTML1 showing predicted protein-coding sequences drawn above or below the DNA line to indicate direction, with predicted biosynthesis domains colour-coded as listed in the key. Modules can be identified by segments of megaproteins running from a KS (red) to an ACP (green). Putative protein binding sites are shown as red and purple discs (in the replication origin, *oriV*) and green discs (‘IR’ for inverted repeat, associated with the biosynthetic cluster promoter regions and likely to be transcriptional regulator binding sites). Like the mupirocin biosynthetic genes the thiomarinol synthases belong to the trans-AT synthases that encode a separate Acyl Transferase while linked to each KS domain is an adjacent “docking domain” consisting of incomplete motifs from Acyl Transferases [11] that may facilitate or regulate acyl transfer [12].

There are a number of errors in Figure 4. Please view the correct Figure 4 here.

**Figure 4:**
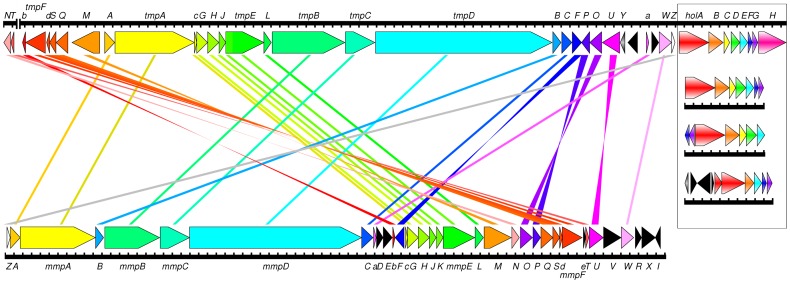
Comparison of the organisation of the thiomarinol gene cluster (upper line) with the mupirocin biosynthesis gene cluster from *Pseudomonas fluorescens*(lower line) and (boxed, on right) with related NRPS clusters from (top to bottom)*Yersinia ruckeri* ATCC29473, *Streptomyces clavuligerus* ATCC27064 and*Photorhabdus asymbiotica* ATCC43949. Lines connecting orfs are simply to help identify equivalent genes and do not indicate the degree of relatedness. A full map of pTML1 is shown in Figure 2. *macpE* (labelled “e”), which is critically missing from the thiomarinol cluster, lies between *mmpF* and *mupT*.

In Table S1, the gene names at coordinates 78712..79242 and 79587..80669 are incorrectly switched. The gene name at coordinate 78712..79242 should be *tmlY*, and the gene name at coordinate 79587..80669 should be *tmuA*. Please view the correct Table S1 here.

## Supporting Information

Table S1 **Predicted gene products of pTML1.**
(DOC)
