# Supplementary material for: A Natural Plasmid Uniquely Encodes Two Biosynthetic Pathways Creating a Potent Anti-MRSA Antibiotic
Source: PLoS One. 2011 Mar 31;6(3):e18031. doi: 10.1371/journal.pone.0018031 (PMC3069032; doi:10.1371/journal.pone.0018031)
Supplement: Table S1 — Predicted gene products of pTML1. (DOC) [file pone.0018031.s002.doc]

**Table S1. Predicted gene products of pTML1.**

| Coordinates (top strand >; bottom < strand) | Gene name | Amino acids number | Molecular weight (kDa) | Predicted function | Nearest relative (protein accession, name and organism): score combines % identity and proportion aligned | % Amino Acid Identity  (% identity to Mup equiv^nt^)^a^ |
| --- | --- | --- | --- | --- | --- | --- |
| 19..798 < | *tmlN* | 259 | 30.691 | Phosphopantetheinyl transferase | [ZP_01465301.1](http://www.ncbi.nlm.nih.gov/entrez/query.fcgi?cmd=Retrieve&db=Protein&list_uids=115378125&dopt=GenPept&RID=U4V1UJRX01N&log$=protalign&blast_rank=1) MtaA *Stigmatella aurantiaca* | 38 (40) |
| 828..1175 < | *tmlT* | 115 | 12.664 | Ferredoxin dioxygenase | [01224340.1](http://www.ncbi.nlm.nih.gov/entrez/query.fcgi?cmd=Retrieve&db=Protein&list_uids=90416409&dopt=GenPept&RID=U4VHSE1R01S&log$=protalign&blast_rank=1) marine gamma proteobacterium | 40 (39) |
| 2420..3430 < | *intA* | 336 | 38.452 | similar to phage integrase | [YP_003494792.1](http://www.ncbi.nlm.nih.gov/entrez/query.fcgi?cmd=Retrieve&db=Protein&list_uids=290243122&dopt=GenPept&RID=U4W6WJHJ01N&log$=protalign&blast_rank=1) *Thioalkalivibrio* sp. | 33 |
| 4322..4687 < | *orfA* | 121 | 14.130 | N-terminal of transposase | ZP_01074710.1 *Marinomonas* sp. | 63 |
| 4961..6091 < | *repA* | 376 | 43.017 | Plasmid replication initiator | [YP_342043.1](http://www.ncbi.nlm.nih.gov/entrez/query.fcgi?cmd=Retrieve&db=Protein&list_uids=77362469&dopt=GenPept&RID=U6CHP5U7014&log$=protalign&blast_rank=1) RepA *Pseudoalteromonas haloplanktis* | 47 |
| 7819..9024 > | *parA* | 401 | 45.708 | ATPase, partitioning | [AAW51293.1](http://www.ncbi.nlm.nih.gov/entrez/query.fcgi?cmd=Retrieve&db=Protein&list_uids=57472466&dopt=GenPept&RID=U6CVC76101N&log$=protalign&blast_rank=1) ParA *Vibrio cholerae* | 74 |
| 9025..10089 > | *parB* | 354 | 40.517 | DNA binding, partitioning | NP_720424.1 ParB *Shewanella oneidensis* | 48 |
| 10748..11083 < | *tacpB* | 111 | 12.656 | Acyl carrier protein | [AAM12918.1](http://www.ncbi.nlm.nih.gov/entrez/query.fcgi?cmd=Retrieve&db=Protein&list_uids=20150016&dopt=GenPept&RID=U6E5NDG901S&log$=protalign&blast_rank=1) mAcp13/B *Pseudomonas fluorescens* | 31 |
| 11086..13338 < | *tmpF* | 750 | 82.731 | Ketosynthase domain | [AAM12934.1|](http://www.ncbi.nlm.nih.gov/entrez/query.fcgi?cmd=Retrieve&db=Protein&list_uids=20150032&dopt=GenPept&RID=U6EX1R3R012&log$=protalign&blast_rank=1) MmpVI/F *Pseudomonas fluorescens* | 43 |
| 13493..13828 < | *tacpD* | 111 | 12.257 | Acyl carrier protein | [AAM12933.1](http://www.ncbi.nlm.nih.gov/entrez/query.fcgi?cmd=Retrieve&db=Protein&list_uids=20150031&dopt=GenPept&RID=U6F4XV5C016&log$=protalign&blast_rank=1) mAcp15/D *Pseudomonas fluorescens* | 46 |
| 13822..14547 < | *tmlS* | 241 | 25.951 | 3-oxoacyl-ACP reductase | [AAM12932.1](http://www.ncbi.nlm.nih.gov/entrez/query.fcgi?cmd=Retrieve&db=Protein&list_uids=20150030&dopt=GenPept&RID=U6FBGN1R01N&log$=protalign&blast_rank=1) MupS *Pseudomonas fluorescens* | 62 |
| 14561..15898 < | *tmlQ* | 445 | 50.181 | Acyl-CoA synthase | [AAM12931.1](http://www.ncbi.nlm.nih.gov/entrez/query.fcgi?cmd=Retrieve&db=Protein&list_uids=20150029&dopt=GenPept&RID=U6FKF0H301N&log$=protalign&blast_rank=1) MupQ *Pseudomonas fluorescens* | 46 |
| 16377..19523 < | *tmlM* | 1048 | 120.816 | Isoleucyl tRNA synthase | ZP_01873397.1 IleS *Lentisphaera araneosa* | 63 (40) |
| 20098..21222 > | *tmlA* | 374 | 42.002 | FMNH(2)-dependent oxygenase | [YP_632117.1](http://www.ncbi.nlm.nih.gov/entrez/query.fcgi?cmd=Retrieve&db=Protein&list_uids=108761496&dopt=GenPept&RID=U6MTHD47016&log$=protalign&blast_rank=1) LuxA *Myxococcus xanthus* | 59 (58) |
| 21265..30297 > | *tmpA* | 3010 | 330.968 | Multifunctional PKS | [AAM12909.1](http://www.ncbi.nlm.nih.gov/entrez/query.fcgi?cmd=Retrieve&db=Protein&list_uids=20150007&dopt=GenPept&RID=U6YNBBXU014&log$=protalign&blast_rank=1) MmpI/A *Pseudomonas fluorescens* | 47 |
| 30294..30560 > | *tacpC* | 88 | 9.822 | Acyl carrier protein | [ZP_05543654.1](http://www.ncbi.nlm.nih.gov/entrez/query.fcgi?cmd=Retrieve&db=Protein&list_uids=256818639&dopt=GenPept&RID=U6Z2GSND016&log$=protalign&blast_rank=1) ACP *Streptomyces griseoflavus* | 46 (45) |
| 30562..31779 > | *tmlG* | 405 | 42.716 | 3-oxoacyl-ACP synthase | ADD82943.1 BatB  *Pseudomonas fluorescens* | 55 (55) |
| 31803..33068 > | *tmlH* | 421 | 46.706 | HMG-CoA synthase | [AAM12922.1](http://www.ncbi.nlm.nih.gov/entrez/query.fcgi?cmd=Retrieve&db=Protein&list_uids=20150020&dopt=GenPept&RID=U6ZPVTBM016&log$=protalign&blast_rank=1) MupH *Pseudomonas fluorescens* | 68 |
| 33091..33858 > | *tmlJ* | 255 | 28.950 | Enoyl-CoA hydratase | [YP_001421290.1](http://www.ncbi.nlm.nih.gov/entrez/query.fcgi?cmd=Retrieve&db=Protein&list_uids=154686129&dopt=GenPept&RID=U6ZXM021012&log$=protalign&blast_rank=1) ECH *Bacillus amyloliquefaciens* | 50 (49) |
| 33858..38192 > | *tmpE* | 1444 | 159.850 | Enoyl-CoA hydratase and ketosynthase | [AAM12925.1](http://www.ncbi.nlm.nih.gov/entrez/query.fcgi?cmd=Retrieve&db=Protein&list_uids=20150023&dopt=GenPept&RID=U707XHCU01S&log$=protalign&blast_rank=1) MmpV/E *Pseudomonas fluorescens* | 40 |
| 38189..39022 > | *tmlL* | 277 | 31.192 | Putative hydrolase | [AAM12926.1](http://www.ncbi.nlm.nih.gov/entrez/query.fcgi?cmd=Retrieve&db=Protein&list_uids=20150024&dopt=GenPept&RID=U70G4RJG01N&log$=protalign&blast_rank=1) MupL *Pseudomonas fluorescens* | 43 |
| 39136..47388 > | *tmpB* | 2750 | 302.629 | Multifunctional PKS | YP_002773469.1 PKS *Brevibacillus brevis* | 37 (36) |
| 47452..50805 > | *tmpC* | 1117 | 123.047 | *Trans* acyl-transferase plus putative enoyl reductase | [AAM12912.1](http://www.ncbi.nlm.nih.gov/entrez/query.fcgi?cmd=Retrieve&db=Protein&list_uids=20150010&dopt=GenPept&RID=U712HJ4V016&log$=protalign&blast_rank=1) MmpIII /C *Pseudomonas fluorescens* | 50 |
| 50878..70959 > | *tmpD* | 6693 | 741.720 | Multifunctional PKS | AAM12913.1 MmpIV/D *Pseudomonas fluorescens* | 38 |
| 71058..71981 > | *tmlB* | 307 | 33.961 | 3-oxoacyl-ACP synthase | AAM12910.1 MupB *Pseudomonas fluorescens* | 38 |
| 72078..73217 > | *tmlC* | 379 | 41.787 | NADH: flavin oxidoreductase | [AAM12914.1](http://www.ncbi.nlm.nih.gov/entrez/query.fcgi?cmd=Retrieve&db=Protein&list_uids=20150012&dopt=GenPept&RID=U7DB7NY501S&log$=protalign&blast_rank=1) MupC *Pseudomonas fluorescens* | 56 |
| 73267..74319 > | *tmlF* | 350 | 38.390 | Ketoreductase | [AAM12919.1](http://www.ncbi.nlm.nih.gov/entrez/query.fcgi?cmd=Retrieve&db=Protein&list_uids=20150017&dopt=GenPept&RID=U7DKEFYX01N&log$=protalign&blast_rank=1) MupF *Pseudomonas fluorescens* | 34 |
| 74316..75236 < | *tmlP* | 306 | 34.830 | Unknown | AAM12930.1 MupP *Pseudomonas fluorescens* | 40 |
| 75246..76598 < | *tmlO* | 450 | 50.999 | Cytochrome P450 | [AAM12929.1](http://www.ncbi.nlm.nih.gov/entrez/query.fcgi?cmd=Retrieve&db=Protein&list_uids=20150027&dopt=GenPept&RID=U7F8NZ62012&log$=protalign&blast_rank=1) MupO *Pseudomonas fluorescens* | 44 |
| 76680..78644 < | *tmlU* | 654 | 72.544 | Acyl-CoA synthase | [NP_252549.1|](http://www.ncbi.nlm.nih.gov/entrez/query.fcgi?cmd=Retrieve&db=Protein&list_uids=15599055&dopt=GenPept&RID=U7FE1PFP012&log$=protalign&blast_rank=1) *Pseudomonas aeruginosa* | 39 (26) |
| 78712..79242 < | *tmuA* | 176 | 19.519 | Membrane protein / Permease | YP_003467672.1 *Xenorhabdus bovienii* | 57 |
| 79587..80669 < | *tmlY* | 360 | 41.154 | Unknown | [ZP_01132443.1](http://www.ncbi.nlm.nih.gov/entrez/query.fcgi?cmd=Retrieve&db=Protein&list_uids=88857800&dopt=GenPept&RID=U7G8EFRD016&log$=protalign&blast_rank=1) *Pseudoalteromonas tunicata* | 49 |
| 81681..81962 > | *tacpA* | 93 | 10.370 | Acyl carrier protein | [AAM12915.1|](http://www.ncbi.nlm.nih.gov/entrez/query.fcgi?cmd=Retrieve&db=Protein&list_uids=20150013&dopt=GenPept&RID=UFETD9R2016&log$=protalign&blast_rank=1) mAcp12/A *Pseudomonas fluorescens* | 51 |
| 82295..83089 > | *tmuB* | 264 | 29.542 | Putative dioxygenase | [ZP_01034759.1](http://www.ncbi.nlm.nih.gov/entrez/query.fcgi?cmd=Retrieve&db=Protein&list_uids=85703655&dopt=GenPept&RID=U7GGEGV2016&log$=protalign&blast_rank=1) *Roseovarius sp.* | 41 |
| 83143..84546 > | *tmlW* | 467 | 54.143 | Dioxygenase (Rieske 2Fe-2S protein) | [AAM12939.1](http://www.ncbi.nlm.nih.gov/entrez/query.fcgi?cmd=Retrieve&db=Protein&list_uids=20150037&dopt=GenPept&RID=U7GTZ7J6012&log$=protalign&blast_rank=1) MupW *Pseudomonas fluorescens* | 56 |
| 84558..84941 > | *tmlZ* | 127 | 14.688 | Chromosomal replication initiation protein | [ZP_01876007.1|](http://www.ncbi.nlm.nih.gov/protein/149198966?report=genbank&log$=protalign&blast_rank=1&RID=GZ6VNH1501N) *Lentisphaera araneosa* | 40 |
| 85410..88778 > | *holA* | 1122 | 125.101 | Non-Ribosomal Peptide Synthase | [YP_003467658.1](http://www.ncbi.nlm.nih.gov/entrez/query.fcgi?cmd=Retrieve&db=Protein&list_uids=290474778&dopt=GenPept&RID=U9D27M4Y012&log$=protalign&blast_rank=1) *Xenorhabdus bovienii* | 55 |
| 88790..90415 > | *holB* | 541 | 58.826 | Oxidoreductase | [YP_003467657.1](http://www.ncbi.nlm.nih.gov/entrez/query.fcgi?cmd=Retrieve&db=Protein&list_uids=290474777&dopt=GenPept&RID=U9D9ZZ32012&log$=protalign&blast_rank=1) flavoprotein *Xenorhabdus bovienii* | 59 |
| 90517..91155 > | *holC* | 212 | 23.971 | Thioesterase | [YP_003467656.1](http://www.ncbi.nlm.nih.gov/entrez/query.fcgi?cmd=Retrieve&db=Protein&list_uids=290474776&dopt=GenPept&RID=U9DJEBVH014&log$=protalign&blast_rank=1) Acyl-CoA hydrolase *Xenorhabdus*  *bovienii* | 42 |
| 91327..92310 > | *holD* | 327 | 35.279 | Acyl-CoA dehydrogenase | [ZP_04616955.1](http://www.ncbi.nlm.nih.gov/entrez/query.fcgi?cmd=Retrieve&db=Protein&list_uids=238755619&dopt=GenPept&RID=U9DUAWM001S&log$=protalign&blast_rank=1) Acyl-CoA dehydrogenase FadE20 *Yersinia ruckeri* | 63 |
| 92330..93214 > | *holE* | 294 | 32.976 | Acetyltransferase | [YP_003042202.1](http://www.ncbi.nlm.nih.gov/entrez/query.fcgi?cmd=Retrieve&db=Protein&list_uids=253990846&dopt=GenPept&RID=U9FD20J5016&log$=protalign&blast_rank=1) *Photorhabdus asymbiotica* | 45 |
| 93255..93719 > | *holF* | 154 | 17.826 | Truncated Globin/ Monooxygenase | [YP_003467654.1](http://www.ncbi.nlm.nih.gov/entrez/query.fcgi?cmd=Retrieve&db=Protein&list_uids=290474774&dopt=GenPept&RID=U9FNB3TN014&log$=protalign&blast_rank=1) *Xenorhabdus bovienii* | 70 |
| 93773..94363 > | *holG* | 196 | 21.417 | Decarboxylase; flavoprotein | [YP_003467652.1](http://www.ncbi.nlm.nih.gov/entrez/query.fcgi?cmd=Retrieve&db=Protein&list_uids=290474772&dopt=GenPept&RID=U9G7V70Z012&log$=protalign&blast_rank=1) Phosphopantothenoylcysteine decarboxylase (flavoprotein) *Xenorhabdus*  *bovienii* | 72 |
| 94447..97557 > | *holH* | 1036 | 113.160 | Unknown | [YP_633455.1](http://www.ncbi.nlm.nih.gov/entrez/query.fcgi?cmd=Retrieve&db=Protein&list_uids=108763128&dopt=GenPept&RID=U9GH0G8M014&log$=protalign&blast_rank=1) *Myxococcus xanthus* | 46 |

a. A % is included in brackets when the first BLAST hit is not the Mup equivalent.
